# Imaging on the Edge: Mapping Object Corners and Edges with Stereo X-Ray Tomography

**DOI:** 10.3390/tomography11080084

**Published:** 2025-07-29

**Authors:** Zhenduo Shang, Thomas Blumensath

**Affiliations:** Institute of Sound and Vibration Research, University of Southampton, Southampton SO17 1BJ, UK; zs4n17@soton.ac.uk

**Keywords:** feature detection, 3D mapping, X-ray image reconstruction

## Abstract

**Background/Objectives:** X-ray computed tomography (XCT) is a powerful tool for volumetric imaging, where three-dimensional (3D) images are generated from a large number of individual X-ray projection images. However, collecting the required number of low-noise projection images is time-consuming, limiting its applicability to scenarios requiring high temporal resolution, such as the study of dynamic processes. Inspired by stereo vision, we previously developed stereo X-ray imaging methods that operate with only two X-ray projections, enabling the 3D reconstruction of point and line fiducial markers at significantly faster temporal resolutions. **Methods:** Building on our prior work, this paper demonstrates the use of stereo X-ray techniques for 3D reconstruction of sharp object corners, eliminating the need for internal fiducial markers. This is particularly relevant for deformation measurement of manufactured components under load. Additionally, we explore model training using synthetic data when annotated real data is unavailable. **Results:** We show that the proposed method can reliably reconstruct sharp corners in 3D using only two X-ray projections. The results confirm the method’s applicability to real-world stereo X-ray images without relying on annotated real training datasets. **Conclusions:** Our approach enables stereo X-ray 3D reconstruction using synthetic training data that mimics key characteristics of real data, thereby expanding the method’s applicability in scenarios with limited training resources.

## 1. Introduction

X-ray computed tomography (XCT) is an established volumetric imaging technique used throughout medical, scientific, and industrial applications. However, in order to generate volumetric images of high spatial resolution and with limited artefacts, the method requires a very large number of individual X-ray measurements to be collected from around the object. Whilst the use of advanced algorithms, such as those based on machine learning or regularised optimisation (e.g., using total variation (TV) constraints), offers the ability to reduce the number of required measurements somewhat [1,2,3,4,5,6,7,8], a significant reduction in the number of measurements is still not possible for most objects without sacrificing image quality.

We recently demonstrated a different approach [9]. Instead of trying to reconstruct full tomographic images from limited observations, which requires very strong prior information, we instead developed a novel stereo X-ray imaging approach that only recovers the 3D location of simple features, such as points and lines. The advantage of our new method is that it only requires two (stereo) projection images but works without the strong constraints that are imposed in full image reconstruction from limited measurements. Our approach thus worked for general objects, as long as these included simple linear fiducial markers. This approach is of particular interest in time-sensitive applications, where the internal structure of an object changes rapidly, and where we might only be able to take a single pair of images at each time step.

In this paper, we extend the above approach from fiducial markers to another commonly found set of linear features, namely, the corners and edges of objects. The location of these point and line features is often more complex to identify. There are several applications where this might be of interest but where fiducial markers cannot be embedded into the object. Of particular interest to us are applications where we want to study a manufactured component with sharp corners and edges whilst they are undergoing rapid deformations.

As in our previous work, we assume an imaging setup with two or more X-ray sources and detectors, providing an X-ray stereo vision system. Using ideas from computer stereo vision, spatial mapping of object corners and edges then becomes possible at the speed of the detector frame rate, which is orders of magnitude faster than full computed tomography data acquisition.

In contrast to visible light imaging, where the light registered at a specific location on a camera’s image sensor is commonly associated with light reflecting from a single 3D point on the surface of an opaque object, X-ray intensity measured on an X-ray detector contains X-ray attenuation information from an entire line through the object [10]. Whilst the main challenge in traditional stereo vision lies in accurately aligning points between the two images forming a stereo pair [11], for X-ray stereo imaging, not only does this matching step become more difficult; an additional challenge is the identification of the location of distinct 3D points in the 2D projected images [9].

### 1.1. Our Method

Our previous work [9] identified an approach that (1) identifies all point-like features in the two X-ray views and (2) matches these features between the views. Once identified and matched, mapping the features into 3D space then employs the same mathematical theory of projective geometry used in traditional stereo vision. To limit computational complexity and allow for efficient scaling to realistically sized X-ray projections, we use a block-based deep learning approach to identify the projected locations of 3D features in 2D space (that is, we identify the features in the projection images). These identified locations are then mapped into 3D space using the filtered back-projection (FBP) method [12,13]. Potential 3D feature locations can then be identified as those points where individual back-projected features overlap between the two views in 3D space. Whilst this is unique, with a high probability if the features are sparse and randomly distributed in space, in order to further enhance robustness against the exact localisation of features on the two imaging planes, we here employ a second deep neural network to process the back-projected volumetric image to identify feature locations.

### 1.2. Contributions

Our previous approach is here extended to the mapping of corner and edge features. Whilst these features are conceptually similar to point and line fiducial markers, the difference is that they are more difficult to identify in X-ray projection images. Whilst using a fiducial marker with a higher X-ray attenuation value will produce projection images with discontinuous image intensities at the fiducial locations, this is not true for edges and corners, where the image intensity in the projected images changes smoothly at the feature locations. Therefore, detecting and matching in this case can pose greater difficulty. Not only do we demonstrate in this paper that our previous approach still works in this setting; we here make a second key contribution. In our previous paper, we used a machine learning method for feature identification that required training. In real applications, we do, however, seldom have sufficient amounts of real training data to train a model for a specific imaging task. To overcome this challenge, we here demonstrate that model training can also be carried out on simplified simulated data matched specifically to a given real imaging setting. Whilst the trained model in this case would not generalise well to other objects, this approach allows more efficient training for a more limited set of object features of interest.

## 2. Methodology

Our methodology uses three key steps: feature detection in each of the two 2D projection images, matching features, and mapping features’ locations in three dimensions.

**Figure 1:**
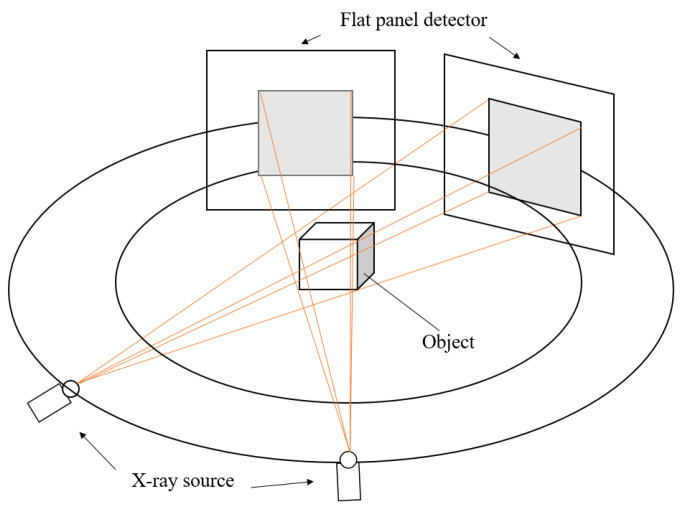
For stereo X-ray tomographic imaging with two views, two X-ray projection images of an object are taken from two different viewing directions.

**Figure 2:**
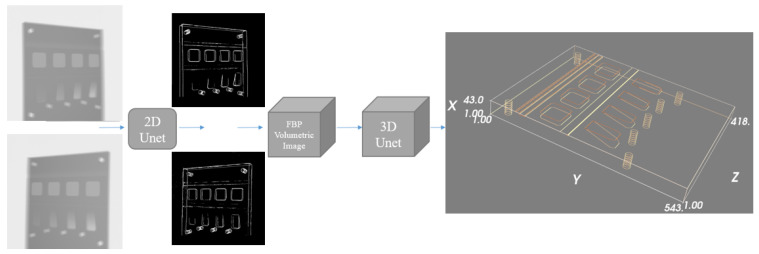
Overview of the proposed framework. The input is a pair of X-ray projection images. Each projection is fed independently into the same 2D U-net to compute two different feature maps, where the background is removed, leaving estimates of the line and point feature locations. Utilising scan geometry knowledge, the two feature maps are then back-projected into a 3D volume using the FDK algorithm, with the back-projected volume further processed using a 3D U-net to generate the 3D spatial feature maps.

The approach utilises a similar point identification and mapping process to our previous work [9]. We assume a stereo X-ray tomography system as shown in Figure 1. Features in each of the stereo images are identified and mapped into 3D space, where the back-projected volume is used to identify 3D feature location. We summarise our proposed approach in Figure 2.

### 2.1. Feature Detection

We formulate the feature detection problem as a standard binary classification problem using a deep neural network that, for each pixel, estimates the probability that this pixel comes from a feature. A neural network implements a parametrised map

(1) $$y_{\mathrm{mask}}=f\left(x_{\mathrm{raw}};\theta \right),$$

where θ are the model parameters, $x_{\mathrm{raw}}\in R^{m\times n}$ are the X-ray projection images encoding the spatial distribution of measured X-ray attenuation on one of the imaging planes, and $y_{\mathrm{mask}}\in B^{m\times n}$ is a pixel-wise class probability map that can be thresholded to estimate feature locations. Parameters θ are adapted using stochastic gradient optimisation to minimise the empirical risk over a training data set ${\left\{\left(x_{\mathrm{raw}}^{i},y_{\mathrm{mask}}^{i}\right)\right\}}_{i=1}^{N}$, which comprises *N* image pairs. The function f(·) in Equation (1) is realised using the standard U-net architecture described in [14] but implemented and trained as a classification network (i.e., using a sigmoid activation function and a binary cross-entropy loss).

### 2.2. Feature Matching and 3D Mapping

As in [9], to derive a robust feature matching and 3D mapping approach, we use standard filtered back-projection methods to map the identified feature locations into 3D space. This is followed by a feature location identification step, where a deep neural network is applied to the 3D image to identify feature locations. Formally, if $B_{L}\left(\cdot \right)$ and $B_{R}\left(\cdot \right)$ are the filtered back-projection operators [15] for the left and right projection images (note—extensions to settings with three or more projections follow the same ideas), then we train a mapping g(·) that maps the two extracted feature maps ${\hat{y}}_{\mathrm{mask}}^{L}$ and ${\hat{y}}_{\mathrm{mask}}^{R}$ to a 3D tomographic volume $y_{\mathrm{vol}}\in R^{m\times n\times o}$.

(2) $$y_{\mathrm{vol}}=g\left(B_{L}\left({\hat{y}}_{\mathrm{mask}}^{L}\right)+B_{R}\left({\hat{y}}_{\mathrm{mask}}^{R}\right);\alpha \right)$$

In this formulation, ${\hat{y}}_{\mathrm{mask}}^{R}$ and ${\hat{y}}_{\mathrm{mask}}^{L}$ are the estimated 2D feature maps from the left and right X-ray images, and $y_{\mathrm{vol}}$ is the estimated volumetric image encoding the probability that each voxel contains one of the features. The function g(·) in Equation (2) is parameterised by trainable parameters α. We use the 3D U-net described in [16] to implement this function.

## 3. Dataset

**Figure 3:**
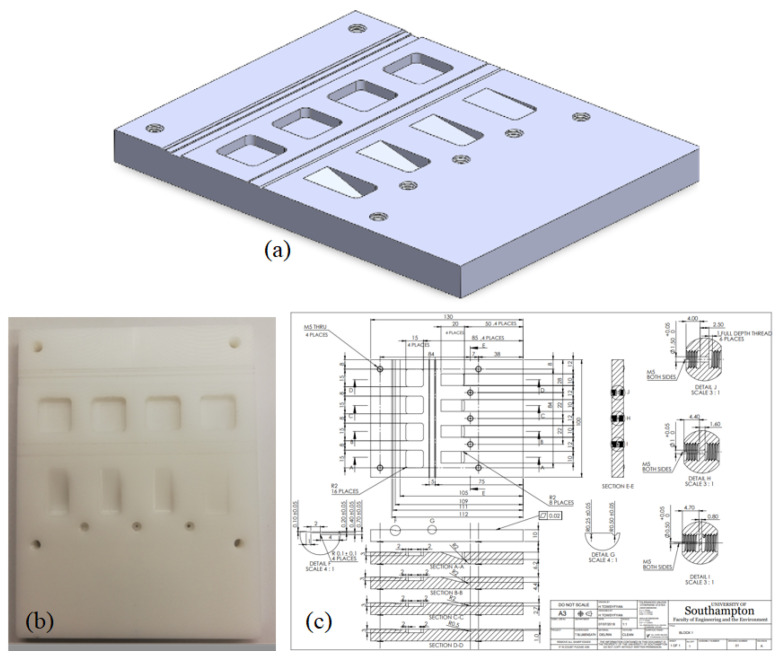
(**a**) A 3D rendering of the 3D block; (**b**) a photo of the workpiece; and (**c**) its CAD drawing.

We demonstrate the method’s ability by mapping the edge and line features of a test phantom manufactured from homopolymer acetal. This phantom was imaged previously in unrelated work [17] but is a useful example here as it contains a range of different linear edge features. We show a photo of the object together with its original design drawing and a 3D rendering in Figure 3.

**Figure 4:**
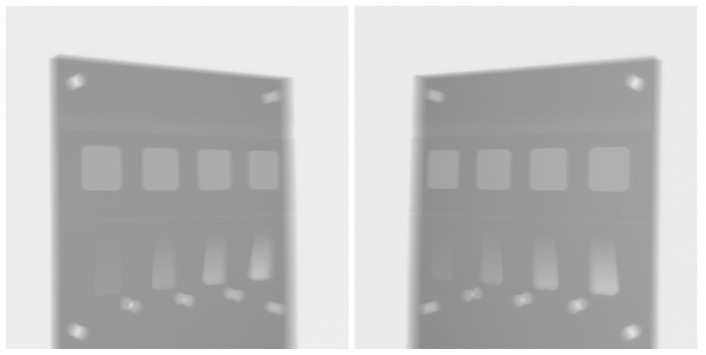
Intensity projection images of the physical test sample, collected by a Nikon XTH225 X-ray tomography system with a 60° relative rotation.

The object was originally scanned over a range of angles, though here we utilise only two projections taken at approximately 60°. Scanning was performed on a Nikon XTH225 X-ray micro-tomography system. The original X-ray intensity images are shown in Figure 4. The 2000×2000 detector had a pixel size of 0.2 mm, and the object was scanned with a source-to-detector distance of about 923 mm and a source object distance of about 290 mm.

**Figure 5:**
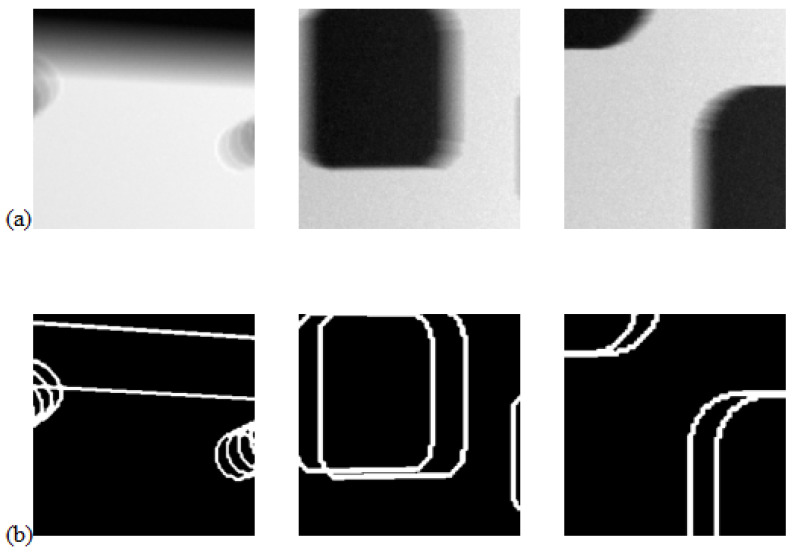
Panel (**a**) shows three projections from the set of training samples used to train the feature detection network, whilst panel (**b**) shows the projected ground truth edge feature maps.

To train the feature detection 2D U-net model, we generated synthetic training datasets, generating 12 3D images of size 256×256×256. Each image contained several simple 3D shapes with straight or rounded corners that approximately matched the shapes expected in the real X-ray images. We then positioned these shapes within a large rectangular prism, assigning low attenuation to the shapes and higher attenuation to the prism. Gaussian noise was added to the background. From these 12 3D images, we generated 24 1024×1024 2D projection images at random object orientations using the Astra Toolbox [18]. Each 2D projection was partitioned into 144 overlapping blocks of size 256×256, providing 3456 samples for training. We show three randomly selected 2D training data pairs in Figure 5, where we show the projection images (top) together with the projected ground truth binary images identifying the locations of the corner and edge features (bottom).

**Figure 6:**
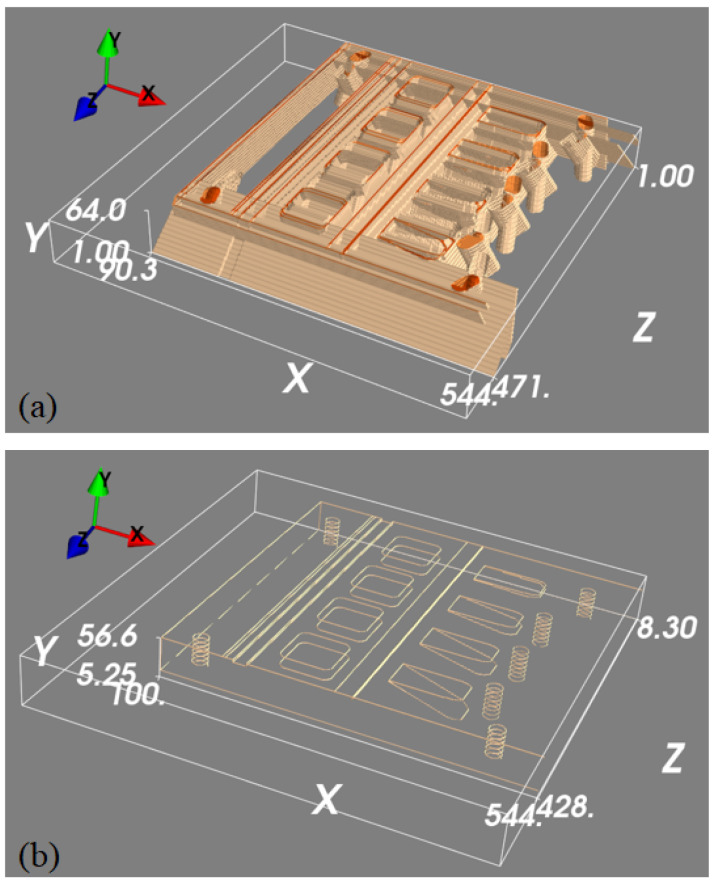
A set of 3D feature mapping training samples. Panel (**a**) is a thresholded 3D rendering of the back-projected volume that is used as the input to the machine learning model, and panel (**b**) is the ground truth we are trying to predict.

To train the 3D U-net to map the detected features in the projection images into 3D space, we generated a 3D edge map from its CAD drawing, which only contains object edge features. To generate a diverse set of images, the same edge feature map was rotated with 1° intervals around an axis parallel to the longest object side. This generated 360 3D images with edge features, each of size 544×64×544 voxels and with a voxel size of 0.24×0.24×0.24 mm^3^. Each of these 3D blocks was then projected to generate pairs of 2D projection images, where projections were collected at ±30°. These ideal 2D feature maps were then back-projected into 3D images using the FDK algorithm to generate simulated back-projected feature maps. When training our 3D U-net, we could thus use the simulated back-projected feature maps as network inputs, with the original clean edge feature maps as desired outputs. Example data is shown in Figure 6.

### Calibration for Stereo X-Ray Imaging System

Whilst we had nominal values for the main system parameters, at the time of scanning, the system was not fully calibrated, so the nominal values given above might have significant errors. Thus, with only two real projections, using the epipolar constraint method [19] with manually selected matching points from two real projections, the relative camera matrix between two cameras could be calculated. We here used the first view as the reference coordinate. Thus, the first camera matrix can be denoted as shown in Equation (3), where $P_{1}$ is the projection matrix of the first view, *K* is the intrinsic matrix, and **I** is the identity matrix. The second camera matrix is denoted as shown in Equation (4), where *R* is the relative rotation matrix and *t* is the normalised translation matrix between two views.

(3) $$P_{1}=K\left[I|0\right]$$

(4) $$P_{2}=K\left[R|t\right]$$

**Figure 7:**
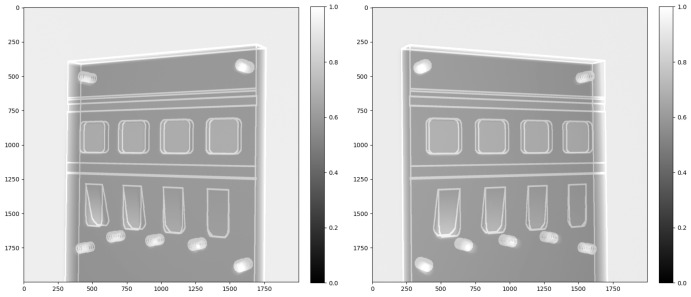
The overlap between simulated edge map projections and the real projections. We measured the geometric error at the object corners and the six screw holes’ positions, finding a five-pixel error on average due to the geometric calibration procedure.

To further refine the calibration, the simulated 3D edge feature map was projected using the two estimated camera matrices and compared visually with the two real projections (see Figure 7). We defined the world coordinates based on the rotation centre of the stereo X-ray imaging system under the Astra Toolbox. Here, we added a tiny pitch, roll and yaw to the centre point of the 3D block outline features to control its pose to make its forward projections under the stereo X-ray geometry mostly overlap with the real projections, while the two viewing angles were −29° and +32°. The comparison of the real outline features and simulated projections is shown in Figure 7, demonstrating a good overlap of the simulated features and the real data after calibration.

## 4. Results

Due to the fact that our system calibration for the available data did not use an optimised and calibrated calibration phantom, errors in the estimated 3D feature location were dominated by calibration errors as well as deviations of the manufactured object’s geometry from the ground truth CAD data (for example, subtractive manufacturing led to a slight warping of the workpiece). Numerical values that tried to quantify the precision of locating features in 3D space were thus dominated by errors in the calibration and assumed ground truth feature locations. Our method is also the first method developed specifically for the 3D mapping of linear X-ray features from stereo X-ray projections, so there is no comparable state-of-the-art method available that we could use for relative performance comparisons. Whilst presenting some numerical results, we primarily limited our evaluation to visual inspection, which was able to show that (a) features were located accurately in the projections and (b) the correct matching was performed in 3D.

We evaluated the feature detection and the 3D feature mapping steps independently.

### 4.1. General Training and Evaluation Approach

Both networks were trained as classification networks for feature detection using the projections (2D network) or the filtered back-projections of the 2D projections (for the 3D network) as inputs and the binary images showing 2D projected or 3D point locations as output. Both networks were implemented using TensorFlow 2.x (TensorFlow, Google Inc., Mountain View, CA, USA) and optimised using an NVIDIA GTX4070ti (NVIDIA Corporation, Santa Clara, CA, USA) graphics card for the 2D network and NVIDIA A100 (NVIDIA Corporation, Santa Clara, CA, USA) graphics cards on the University of Southampton IridisX cluster (Southampton, UK) for the 3D network. We used the Adam optimiser with the synthetic training data, a learning rate of $10^{-4}$, and 100 epochs. The loss function was the binary cross-entropy.

### 4.2. Feature Detection

**Figure 8:**
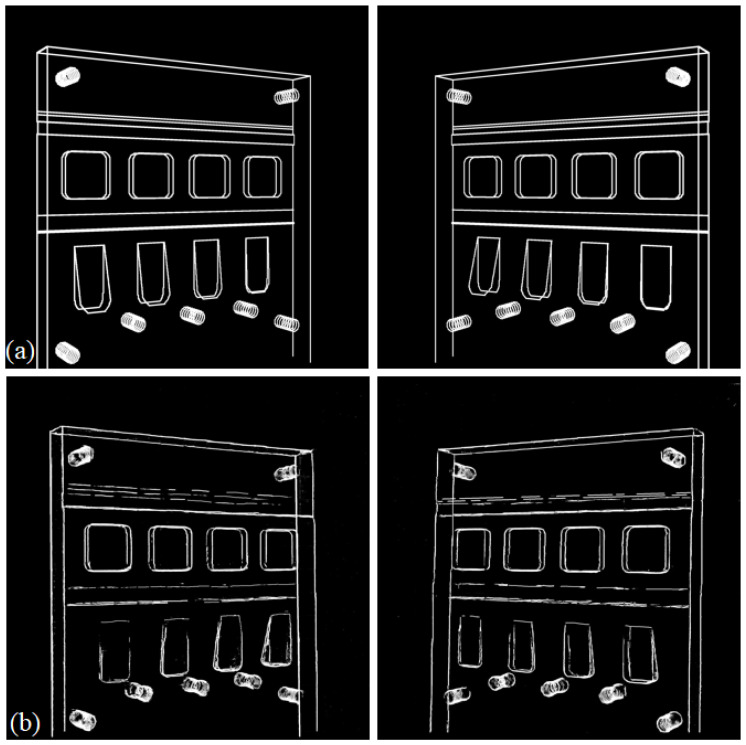
Comparison between the simulated ground truth 2D feature maps (derived from the CAD data of the object) (**a**) and the 2D feature maps estimated from the real data (**b**).

After training the 2D feature detection U-net using the synthetic data described above as the training set, we used the real projection images from the physical phantom to test the method. The real test images were processed by converting the measured X-ray intensity into attenuation values before cropping the images into 338 overlapping blocks of size 256×256. We then applied the 2D U-net to all test sample blocks from both projections. Probabilities were averaged over all blocks that contained a particular image pixel before thresholding to produce a full-size feature map. To evaluate the performance of the method, we used the simulated projections after calibration as our ground truth and visually compared the CAD data as approximate ground truth to the estimated 2D feature maps (See Figure 8).

### 4.3. Three-Dimensional Feature Mapping

We then used the estimated 2D features of the real projections from the feature detection model and, for comparison, the two simulated (CAD-based) feature projections in order to generate two back-projected volumes using the same calibrated projection geometry. Both back-projected volumes went through the 3D model trained for 3D spatial position estimation.

**Figure 9:**
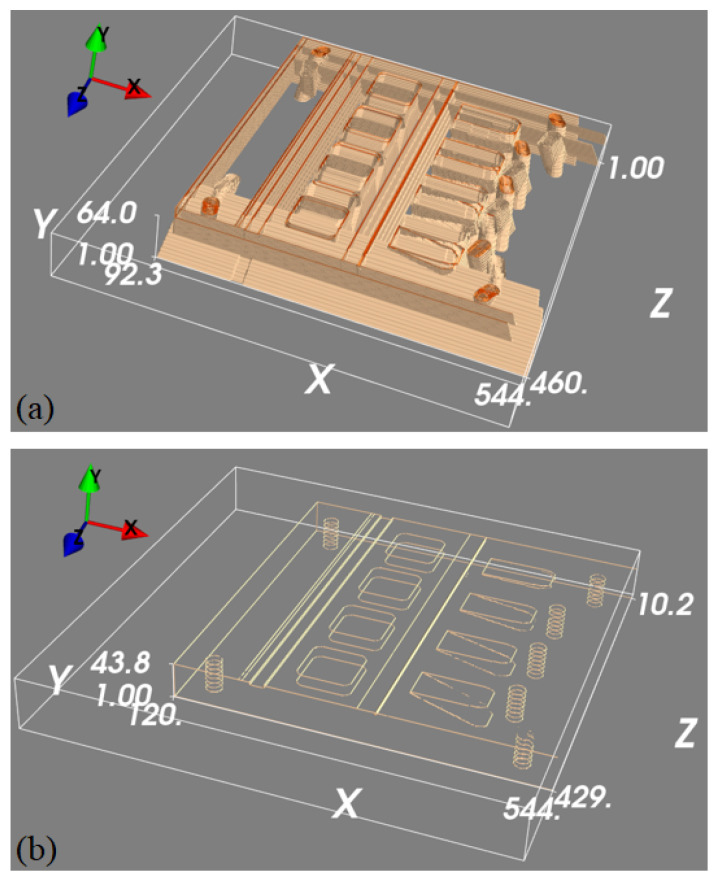
Visualisation of the operation of the 3D U-net. Panel (**a**) shows the back-projected volume generated from two simulated projections of the edge feature maps, whilst panel (**b**) is the 3D edge feature map estimated from the top image using the 3D U-net.

**Figure 10:**
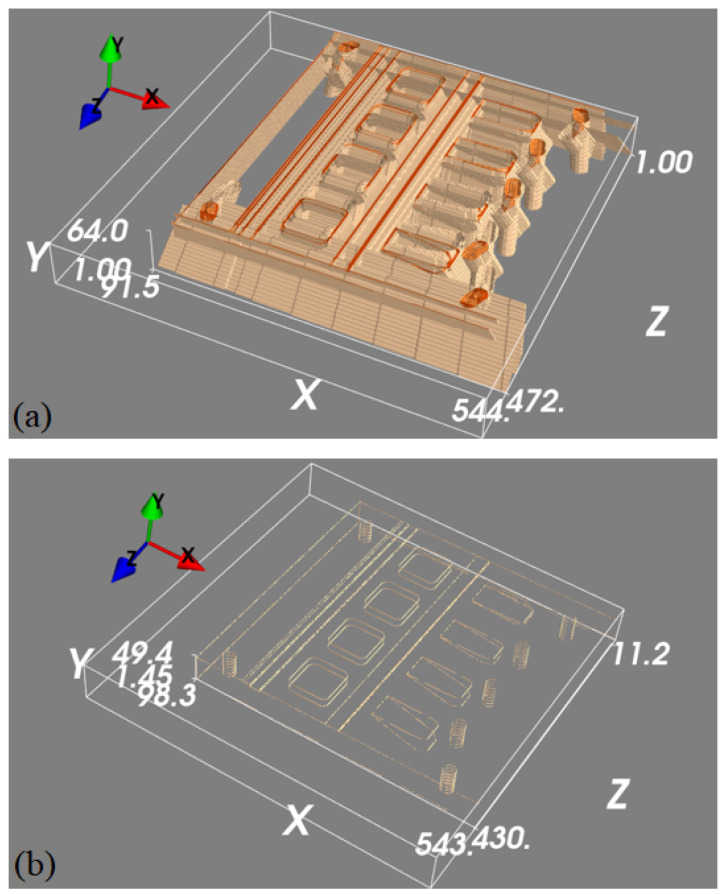
(**a**) Back-projected feature maps and (**b**) estimated 3D features for the real data.

**Figure 11:**
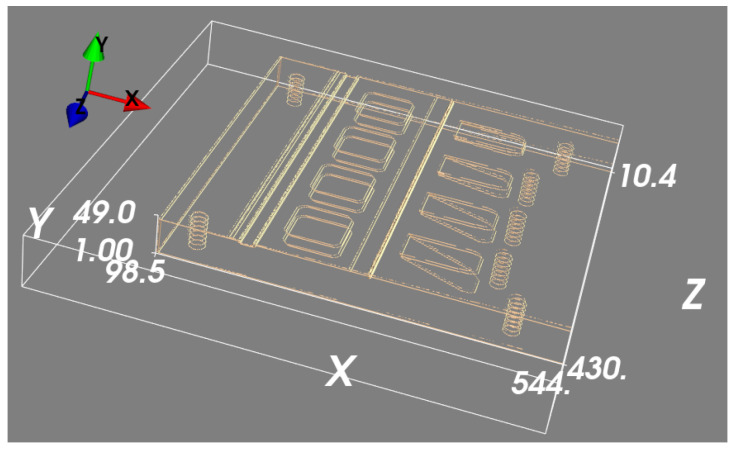
Positioning error in 3D space between the 3D outline estimation by simulated projections and real outline features.

The back-projected volumes generated by the two simulated projections are shown in Figure 9 together with the 3D edge feature map estimated with the 3D U-net model. This can be compared to the back-projected volume generated by the two 2D feature maps estimated from the real data shown in Figure 10, where we also show the 3D edge feature map estimated with the 3D U-net model. To numerically evaluate the error between these two estimations and the ground truth, we compare their corner and screw hole positions to those for the simulated phantom. As seen in Figure 11, the errors from corners and screw holes were in the range of between 1 and 7 voxels, or 0.24 mm to 1.68 mm. Considering the size of the 3D block and the inaccuracy from calibration, these are relatively small errors, which are assumed to be mainly due to the ad hoc post-scan calibration used here. Better calibration results could be obtained using a dedicated calibration object with a fixed stereo X-ray imaging setup.

Our numerical evaluation of the results was based on the location of features in a nominal geometry that was also used for system calibration and should theoretically be aligned with the true object location. Distance errors in feature location between 0.24 mm and 1.68 mm were found, which were likely dominated by errors in the calibration process, though a detailed analysis of the dimensional accuracy of the approach will be left to a future study where a more controlled system calibration approach can be used.

**Figure 12:**
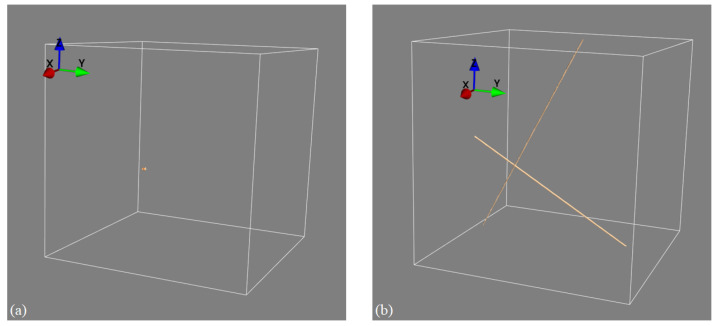
Differences between stereo X-ray tomography and limited-view tomography. (**a**) Stereo XCT reconstruction of a point-like object. (**b**) FBP reconstruction of the same object; the object’s information is smeared out along the X-ray paths that intersect the object.

It is important to contrast our approach to related methods such as limited-angle tomography [20]. In limited-angle tomography, a full 3D object reconstruction is computed from a few projective measurements using advanced iterative or deep learning-based image reconstruction algorithms. This is different from our approach in that our stereo XCT approach only reconstructs the 3D location of point and line features (such as the edges and corners of objects, as described in the current paper). Whilst stereo XCT thus only recovers limited 3D information compared to limited-angle XCT, limited-angle CT has the disadvantage that it requires extremely strong prior knowledge about the imaged object, which is then exploited in the iterative or deep learning-based reconstruction. If we have no prior information on object geometry and density profiles, then limited-angle reconstruction does lead to images with significant artefacts. For example, filtered back-projections computed from a pair of projection images would lead to an image almost without any discernible information. Our stereo XCT method is on the other hand still able to identify object edges and corners, even if we know nothing about the object’s shape and density profile. In fact, edges and corners can always be mapped if we are able to locate and match the edges in the 2D projections without the need to impose any additional prior object knowledge. This is demonstrated in an example where we image a point-like object using two X-ray projection views measured from two viewpoints that are roughly 60 degrees apart. The left panel in Figure 12 shows the recovery of that point when using our stereo XCT technique, whilst the right panel uses the prior information and agnostic filtered back-projection reconstruction, which reconstructs the point as two lines in 3D space. If the X-ray views in addition had contained additional objects without clear edges or corners, then the stereo XCT reconstruction would not have been affected, whilst the limited-angle reconstruction would have been further contaminated by the additional X-ray attenuation estimates from the additional object (but again smeared out along the X-ray paths of the two views).

Our approach also differs substantially from X-ray-based methods proposed in medical imaging that track objects during surgery. Here, a single projection image is often used in which an object, such as a medical instrument or tumour, is identified and then tracked in a single 2D X-ray projection image [21]. Whilst some of these methods also use deep learning-based methods to estimate the location of the object in 3D space, as this information is not measured in a single X-ray projection, this estimate needs to rely again on prior information such as knowledge of typical human anatomy or preoperative full CT scans.

## 5. Discussion and Conclusions

In this paper, we extended the stereo X-ray tomography framework from our previous work to the estimation of the 3D location of the corners and edges of 3D objects. Using two deep neural networks trained using simulated data, we could extract point and line feature locations from two real projection images and, using the calibrated camera matrices of the system, project these back into 3D space. To identify feature location in 3D space, a further 3D neural network was used, again trained on simulated data.

Considering that the main objective of this work was to extend our previous study by applying the stereo X-ray tomography system to more complex and realistic scenarios, we aimed to reconstruct 3D features using only a pair of uncalibrated projection images and the object’s initial blueprint. While the geometry estimated through calibration may introduce certain errors, which in turn affect the accuracy of feature localisation and consequently impact the final 3D mapping, the resulting errors appear to be within a manageable range. From a qualitative perspective, the system successfully achieved its intended purpose and produced reliable results, identifying 2D feature locations with few omissions and false positives, whilst the 3D method clearly matched the correct features to translate these into 3D.

A potential limitation that warrants further investigation in future work is the generalizability of this approach to different feature geometries and object complexities. Although our training data was generated using simple geometric patterns placed in various configurations, there are inherent differences in appearance and physical characteristics from real experimental data. This introduces a possible risk that visual similarities may not fully capture underlying structural differences, potentially affecting generalisation performance. We plan to address this limitation in future work by further enhancing the diversity and realism of the training data. In this work, we roughly categorised features into point features (such as highly attenuating points and corners) and line features (including highly attenuating linear features and object edges). However, in real-world applications, features may often be more difficult to estimate. Point-like and line-like features can potentially vary in their width (for example, a rounded object corner leads to a slightly smeared-out linear feature in the projections). Furthermore, the strength (and thus visibility) of these features in the projection image will vary with object contrast. For a method trained for a specific application, it is thus crucial to match these aspects in the simulated data to the expected feature range in the real data. If instead the aim is to develop a deep learning-based model that works over a much wider range of objects, the greater variation of feature width and contrasts expected in this more general set of applications will need to be taken into account. As always in deep learning-based methods, matching the statistics of these properties in the training data to those in the expected real data will minimise the expected average error.

Herein, we were also limited in that we only had suitable data for a single object. The effect of geometric object complexity, imaging noise, and (possibly) contrast on multi-material objects was thus not evaluated, though the influence of noise on feature detection has already been studied in our previous work with fiducial markers, and it is to be expected that increased noise will lead to decreased performance in 2D feature detection. For the detection of edge features, the ‘sharpness’, i.e., the radius of an object’s edge, will likely also play a key role in the exact localisation of the edge in both two dimensions and, ultimately, three dimensions. These are issues we hope to investigate in future work. 

## Data Availability

The authors confirm that the data supporting the findings of this study are available within the article.

## Abbreviations

The following abbreviations are used in this manuscript:

XCT	X-ray computed tomography
3D	Three-dimensional
FBP	Filtered back-projection
TV	Total variation
